# Physiological Expression of Ion Channel Receptors in Human Periodontal Ligament Stem Cells

**DOI:** 10.3390/cells8030219

**Published:** 2019-03-06

**Authors:** Luigi Chiricosta, Francesca Diomede, Oriana Trubiani, Placido Bramanti, Emanuela Mazzon

**Affiliations:** 1 IRCCS Centro Neurolesi “Bonino-Pulejo”, 98124 Messina, Italy; luigi.chiricosta@irccsme.it (L.C.); placido.bramanti@irccsme.it (P.B.); 2 Department of Medical, Oral and Biotechnological Sciences, University “G. d’Annunzio” Chieti-Pescara, 66100 Chieti, Italy; francesca.diomede@unich.it (F.D.); trubiani@unich.it (O.T.)

**Keywords:** human periodontal ligament stem cell, RNA-seq, transcriptome analysis, next generation sequencing, GABA_B_ receptor, acethylcoline receptor

## Abstract

The etiopathogenesis of neurodegenerative diseases is characterized by the death of neurons. Human periodontal ligament stem cells (hPDLSCs), coming from neuronal crest, can potentially become neuronal cells because of their embryologic origin. In this study, we performed an RNA-seq analysis of hPDLSCs in order to determine whether their transcriptomic profile revealed genes encoded for ion channel receptors. Next, each found gene was enriched by the information of pathways stored in the Reactome database. Our results show that the hPDLSCs express *GABBR1* and *GABBR2, CHRNA1*, *GRINA* genes, respectively associated with GABA_B_, NMDA and nACh receptors. In particular, the two subunits of GABA_B_ receptor are expressed in hPDLSCs. Further, the proteic extract for GABA_B_R1, GABA_B_R2 and AChRα1 confirmed their expression in hPDLSCs. Our results show that hPDLSCs express physiologically genes associated with ion channel receptors maintaining multipotent features which are useful for neurogenesis.

## 1. Introduction

The embryonic head development, together to dental structure formation, is founded on a complex and delicate process directed by a specific, hierarchically-defined genetic program [1]. Teeth are ectodermal appendages whose development is influenced by reciprocal interactions between the surface epithelium (ectoderm) and the underlying neural crest-derived mesenchyme. These interactions are arbitrated by several conserved signaling pathways, such as: Wnt, bone morphogenetic protein (BMP), fibroblast growth factor (FGF), sonic hedgehog (SHH), and ectodysplasin A (EDA) [2]. The neural crest (NC), the embryonic structure present in vertebrates, is indicated as a “fourth germ layer”; it is constituted from multipotent cells, named neural crest cells (NCCs), which are able to differentiate into a surprising array of adult structures. NCCs are a transient multipotent stem cell population that originally appears at the junction of the epidermal and neural ectoderm by reciprocal interactions between these tissues and by signals from the mesoderm [3]. During neurulation process, NCCs begin to delaminate from the dorsal region of the neural epithelium, going toward epithelial-mesenchymal transition [4]. Subsequently, they migrate into their destination in the embryo to generate various groups of cells, including: sensory, autonomic, and enteric ganglia within the peripheral nervous system; and the adrenal medulla, melanocytes, in addition to skeletal, connective, adipose, and endocrine cells [5]. Actually, several studies indicate that NCCs play a crucial role in embryonic development, representing a likely stem cell population that may be an innovative approach in the regeneration of a large repertoire of tissues as well as neuronal cells [6,7,8]. In fact, unfortunately, for most diseases which affect the Central Nervous System (CNS), no treatment exists. When it does exist, it is typically just either a temporary or a palliative solution. For this reason, studies focused on replacing lost neuronal cells by unspecialized cells, like stem cells, are increasing [9,10,11]. Neural crest stem cells could be particularly well-suited to replace nervous system cells. In order to become neuronal cells, they have to express some proteins on their membranes which are able to bind to neurotransmitter molecules like the gamma-Aminobutyric acid (GABA), glutamate or the acetylcholine. These proteins are called neurotransmitter receptors; they are essential for the transmission of signals between cells. Neurotransmitter receptors are classified in ligand-gated ion channels (or ionotropic) and metabotropic receptors. The ionotropic receptors directly allow the flow of ions (Na^+^, K^+^, Ca^2+^, Cl^−^) through the membrane cell when the neurotransmitter binds them. Typical receptors of this class are nicotinic acetylcholine (nAChR), GABA_A_, α-amino-3-hydroxy-5-methyl-4-isoxazolepropionic (AMPA), (2S,3S,4S)-3-(Carboxymethyl)-4-(prop-1-en-2-yl)pyrrolidine-2-carboxylic acid (Kainate), and *N*-methyl-d-aspartate (NMDA) receptors. Typical metabotropic receptors are muscarinic acetylcholine (mAChR), metabotropic glutamate (mGluR), GABA_B_ receptors. When a neurotransmitter saturates a receptor, the associated G protein complex changes conformation, and a secondary message is released. The focus of this paper is to evaluate whether hPDLSCs express physiologically genes encoded for ion channel receptors. Using the Next Generation Sequencing (NGS) technology, our study performed a transcriptomic analysis of a cluster of hPDLSCs from healthy control individuals. The expressed genes were enriched by the information of the cellular pathways in the Reactome database [12].

## 2. Materials and Methods

### 2.1. Cell Isolation

hPDLSCs were isolated from periodontal tissue of three different healthy males of 35–40 years of age. None of them was a smoker. The Medical School “G. d’Annunzio” University, Chieti, Italy, received the approval by the Ethical Committee about cell isolation and culture protocol (number 266/17 April 2014). Before tissue collection, the individuals taking part in the project gave informed consent. The experiments followed the pertinent guidelines and regulations. The hPDLSCs were isolated according to Dominici, M [13]. The surface of the coronal root was scraped using a Gracey’s curette [14]. The undifferentiated cells were then cultured through Mesenchymal Stem Cell Growth Medium Chemically Defined (MSCGM-CD) (Lonza, Basel, Switzerland) and preserved at 37 °C in an incubator with a 5% CO_2_ concentration in the air. 

### 2.2. Cells Culture

The hPDLSCs were cultured according to the method described by Seo B. [15]. They grew in a 25 cm^2^ flask with a MSCGM-CD that was changed every two days. The cells at passage 2 were then used for the following experiments. The morphological evaluation of the cells was made by microscopy DMIL to detect a toluidine blue solution (Leica Microsystem, Milan, Italy) [16]. 

The human dermal fibroblasts (type DPK-SKDF-H), used as a control, were donated by the Department of Biomedical and Dental Sciences and Morphofunctional Images of University of Messina and acquired from Dominion Pharmakine (Bizkaia, Spain). The DPK-SKDF-H were cultured in a Dulbecco’s Minimal Essential Medium (DMEM) to which were added 10% Fetal Bovine Serum (FBS), penicillin/streptomycin (100 units/mL, 100 µg/mL) and L-glutamine (2 mmol/L) inside a 75 cm^2^ plastic flask. They were then incubated at 37 °C in humidified air with 5% CO_2_.

### 2.3. Cytofluorimetric Evaluation

Five lots of 10^5^ hPDLSCs were placed in a 6-well tissue culture plate and incubated with 1 µg of the specific antibody, conjugated with fluorescein isothiocyanate (FITC), phycoerythrin (PE), allophycocyanin (APC), phycoerythrin-cyanine 5.5 (PE Cy5.5), or Alexa Fluor 488 for 30 min at 4 °C in the dark. The hPDLSCs were stained using the following antibodies: anti-CD29, anti-CD44, anti-CD45, anti-CD105 (Ancell, Bayport, MN, USA); anti-CD14 (Milteny Biotec, Bergisch Gladbach, Germany); anti-CD73, anti-CD90, anti-OCT3/4, anti-Sox2 and anti-SSEA4 (Becton Dickinson, BD, San Jose, CA, USA); anti-CD34 (Beckman Coulter, Fullerton, CA, USA). After incubation, the cells were acquired with a flow cytometer (FACSCalibur^TM^; Becton Dickinson, BD, San Jose, CA, USA). Data were analyzed by the FlowJo software v8.8.6 (TreeStar, Ashland, OR, USA). 

### 2.4. MTT Assay

The viability of the hPDLSCs was determined using the 3-(4,5-dimethylthiazolyl-2)-2,5-diphenyltetrazoliumbromide (MTT) method. Two lots of 10^3^ cells/well were placed in a 96-well tissue culture plate and incubated at 37 °C for 24, 48, 72 h and 1 week. At each time point, MTT solution (20 µL) (Promega, Milan, Italy) was added to each well to detect the metabolic activity of the cells [17,18].

### 2.5. Mesengenic Differentiation

The hPDLSCs were induced to osteogenic and adipogenic differentiation commitment, as previously reported by Cianci et al. [19]. Briefly, at the end of differentiation process, the cells were stained with Alizarin Red S (Sigma-Aldrich, Milan, Italy) and Adipo Oil Red (Lonza, Basel, Switzerland) solution to evaluate the osteogenic and adipogenic differentiation, respectively, and observed by means of light microscopy, Leica DMIL (Leica Microsystem, Milan, Italy) [20].

### 2.6. Statistical Analysis

Data were analyzed using GraphPad Prism 6.0 (GraphPad Software, La Jolla, CA, USA). The statistical analyses were performed with the ANOVA test. The *p*-value threshold used to validate the analysis is 0.05.

### 2.7. RNA Isolation and Real Time-PCR Analysis

To assess osteogenic and adipogenic differentiation ability of PDLSCs, the total RNA was isolated using the Total RNA Purification Kit (NorgenBiotek Corp., Ontario, CA, USA) according to the manufacturer’s instructions. The RNA was extracted from a pool of 5 × 10^5^ hPDLSCs. M-MLV Reverse Transcriptase reagents (Applied Biosystems) were used to generate cDNA. Real-Time PCR was carried out with the Mastercycler ep realplex real-time PCR system (Eppendorf, Hamburg, Germany). hPDLSCs expression of Runt-related transcription factor-2 (*RUNX-2*) and AlkalinPhospatase (ALP) was evaluated after 28 days in osteogenic differentiated culture, the expression of Fatty Acid Binding Protein 4 (FABP4) and Peroxisome Proliferator-Activated Receptor γ (PPARγ) were analysed after 28 days of adipogenic differentiation culture. Commercially available TaqMan Gene Expression Assays (*RUNX-2* Hs00231692_m1; ALP Hs01029144_m1; FABP4 Hs01086177_m1; PPARγ Hs01115513_m1) and the TaqMan Universal PCR Master Mix (Applied Biosystems, Foster City, CA, USA) were used according to standard protocols. Beta-2 microglobulin (B2M Hs99999907_m1) (Applied Biosystems, Foster City, CA, USA) was used for template normalization [21]. RT-PCR was performed in three independent experiments; duplicate determinations were carried out for each sample.

### 2.8. Scanning Electron Microscopy

The hPDLSCs at second passage were fixed for 4 h at 4 °C in 4% glutaraldehyde in 0.05 M phosphate buffer (pH 7.4), and subsequently dehydrated in increasing ethanol concentrations and then critical-point dried. Samples were then mounted onto aluminum stubs and gold-coated in an Emitech K550 (Emitech Ltd., Ashford, UK) sputter-coater before imaging by means Zeiss SEM EVO 50 (Zeiss, Jena, Germany) [22].

### 2.9. Immunofluorescence

In order to evaluate the positivity to the neuronal marker, the hPDLSCs were incubated with primary monoclonal antibodies: anti human p75 (1:200, rabbit) (Santa Cruz Biotechnology Inc., Santa Cruz, CA, USA) and nestin (1:200, rabbit) (Santa Cruz Biotechnology Inc., Santa Cruz, CA, USA). As secondary antibody we used Alexa Fluor 568 red fluorescence conjugated (1:200) (Molecular Probes^TM^, Invitrogen^TM^, Eugene, OR, USA). Alexa Fluor 488 phalloidin green fluorescence conjugate (1:200, Molecular Probes^TM^, Invitrogen^TM^, Eugene, OR, USA) was used to stain cytoskeleton actin and TO-PRO-3 (1:200, Molecular Probes^TM^, Invitrogen^TM^, Eugene, OR, USA) to stain nuclei. Stained cells were observed using confocal laser scanning microscopy (LSM800 META, Zeiss, Oberkochen, Germany) [23].

### 2.10. RNA-Seq Preparation

The Reliaprep RNA cell Miniprep System (Promega, USA) was used to extract the total RNA from all of the samples. Each of them was treated with 0.1% of DMSO. The TruSeq RNA Access library kit protocol (Illumina, San Diego, CA, USA) was followed. From the total RNA, 40 ng were extracted and fragmented through a thermal cycler for 8 min at 94 °C. The achieved fragments, >200 nt, were used to synthesize a first strand of cDNA using the activity of the reverse transcriptase SuperScript II (Invitrogen, Carlsbad, CA, USA). The Second Strand Marking Master Mix was used to obtain the double strand of the cDNA. The incubation ran for 1 h at 16 °C. The reaction mix was eliminated by AMPure XP beads purification. Fragment adhenilation was performed at 3′ ends in order to ligate the complementary adapters, thereby avoiding huge chimera development. The identification of the samples and their preparation for flow cell hybridization was performed by the ligation of Adapter-Indexes to the fragment of cDNA. A first step of clean-up was run before the PCR amplification (denaturation: 30 s at 98 °C; 15 cycles: 10 s at 98 °C, 30 s at 60 °C, 30 s at 72 °C; extension: 5 min at 72 °C). The regions of interest are selected and enriched via a first reaction of hybridization (10 min at 95 °C, un minute of incubation per 18 cycles, 90 min at 58 °C). The procedure allowed us to mix the exome capture probes with the cDNA library. Starting from 200 ng of each library, the result is a pool of libraries which were differently indexed. The purification of the pool was obtained by streptavidin conjugated magnetic beads. Then, subsequent hybridization and purification were performed. A second PCR amplification was done following the same protocol as that of the first one, except for the cycles (just 10 instead of 15). The process ended with a clean-up. The Bioanalyzer instrument (Agilent High Sensitivity DNA Kit, Richardson, TX, USA) was used to validate the quality of the library. The validation of the quantity was made using Real-Time PCR (KAPA Library Quantification Kit-Illumina/ABI Prism@) (Kaba Biosystem, Inc. Wilmington, MA, USA). A denaturation step through 2N NaOH was performed, and then it was diluted until it reached a concentration of 12 pM. The MiSeq Instrument (Illumina, San Diego, CA, USA) was used to sequencing (by single read setting) using MiSeq Reagent Kit v3 for 150 cycles.

### 2.11. RNA-seq Data Processing and Gene Analysis

The MiSeq instrument provides multiplexed samples in “bcl” format. The CASAVA software (version 1.8) was used to convert these data in demultiplexed “Fastq” files, one for each sample. Then, the quality of the reads was checked using the fastQC software. The reads were trimmed of the adapters by Trimmomatic [24]. Finally, the reads are aligned to the reference genome “homo sapiens UCSC hg19” using STAR RNA-seq aligner [25]. All the transcripts which were common among the samples were then collected together using Cufflinks [26]. The set of genes that transcribed the found transcripts were used for the study. The whole gene list was inserted as file in the “Analysis” tools section of the Reactome database. From this list, a subset of 64 genes included in the “Neuronal System” pathways were studied. A deeper inspection of each of these genes showed that SAT1 was identified by a UNIPROT [27] entry that is related to *SLC38A1* gene, and *P3H3* is identified by a UNIPROT linked to *GNB3*. Finally, *SAT1*, *P3H3* and *HSPA8* genes are excluded from the analysis. By a literature inspection, were searched for genes encoded for proteins associated with ion channel receptors and present in this list of genes. GABAergic, Glutamatergic and Cholinergic pathway associations were found. The genes in the GABAergic subpathways are expressed in GABAergic “GABA receptor activation” and “GABA synthesis, release, reuptake and degradation”, while for Glutamatergic ones, “Glutamate binding, activation of AMPA receptors and synaptic plasticity”, “Activation of kainate receptors upon glutamate binding”, “Glutamate Neurotransmitter Release Cycle”, “Astrocytic Glutamate-Glutamine Uptake and Metabolism”, “Activation of NMDA receptors and postsynpatic events”, as well for Cholinergic “Acetylcholine Neurotransmitter Release Cycle” and “Activation of Nicotinic Acetylcholine Receptors”.

### 2.12. Western Blot Analysis

The proteins were collected from hPDLSCs as previously described [9]. In parallel, the proteins from DPK-SKDF-H were also collected and used as a control. Protein concentrations were measured using Bradford assay (Bio-Rad Laboratories, Inc., Hercules, CA, USA). Twenty-five µg of proteins were heated for 5 min at 95 °C and resolved by SDS-polyacrylamide gel electrophoresis (SDS-PAGE), and then transferred onto a PVDF membrane (Amersham Hybond, GE Healthcare Life Sciences, Milan, Italy). Membranes were blocked with 5% skim milk in Phosphate Buffered Saline (PBS) for 1 h at room temperature. Afterwards, the membranes were incubated overnight at 4 °C with the following primary antibodies: GABAB R1 (1:500; Santa Cruz Biotechnology, Dallas, TX, USA), GABAB R2 (1:500; Santa Cruz Biotechnology, Dallas, TX, USA) and AChRα1 (1:500; Santa Cruz Biotechnology, Dallas, TX, USA). After washing the membranes with PBS 1X, they were incubated with horse radish peroxidase (HRP)-conjugated anti-mouse or anti-rat IgG secondary antibodies (1:2000; Santa Cruz Biotechnology, Dallas, TX, USA) for 1 h at room temperature. The membranes were also incubated with an antibody for glyceraldehyde 3-phosphate dehydrogenase (GAPDH) HRP Conjugated (1:1000, Cell Signaling Technology, Danvers, USA). The bands were analyzed through an enhanced chemiluminescence system (Luminata Western HRP Substrates; Millipore, Burlington, MA, USA), and the protein bands were acquired through ChemiDoc™ MP System (Bio-Rad Laboratories Inc., Hercules, CA, USA).

## 3. Results

### 3.1. Cytofluorimetric Analysis

**Figure 1:**
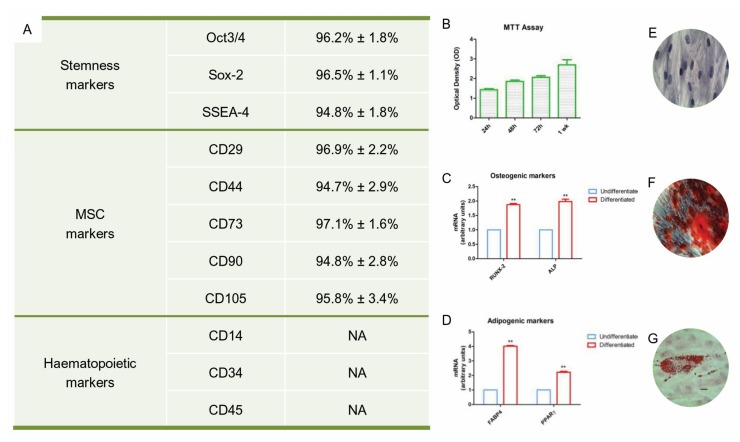
hPDLSCs characterization. (**A**) Flow cytometry of surface related markers at the 2nd passage. (**B**) MTT Graph showed the proliferation rate at different time of culture. (**C**) RT-PCR of osteogenic markers: RUNX2 and ALP. (**D**) RT-PCR of adipogenic markers: FAPB4 and PPARγ. (**E**) Plastic adherent hPDLSCs stained with Toluidine blue solution (**F**) Alizarin Red S staining of hPDLSCs after 28 days of differentiation. (**G**) Oil Red staining of hPDLSCs after 28 days of differentiation. Mag: 10× (**E**); 40× (**F**,**G**), bar: 10 µm. The ANOVA test (*p*-value threshold of 0.05) was used to perform the statistical analysis between osteogenic and adipogenic markers.

The phenotype of the hPDLSCs was confirmed by flow cytometric analysis. The cells showed a positivity for OCT3/4, SSEA4, SOX2, CD29, CD44, CD73, CD90 and CD105. They were negative for haematopoietic markers such as CD14, CD34 and CD45 (Figure 1A).

### 3.2. MTT Assay Analysis

In order to access to the proliferation rate of the hPDLSCs, an MTT assay analysis was performed. The proliferative rate was detected at 24 h, 48 h, 72 h and after 1 week of culture, as shown in the Figure 1B. The proliferation rate grew according to a logarithmic pathway. 

### 3.3. Mesengenic Differentiation and Genetic Evaluation

To evaluate osteogenic differentiation, hPDLSCs were stained with an Alizarin Red S solution. Calcium precipitates were detected in hPDLSCs culture after 28 days of incubation (Figure 1F). The adipogenic differentiation was evaluated using lineage Oil-red O solution staining; cells showed lipid droplets at the cytoplasmic level (Figure 1G). To confirm the osteogenic and adipogenic commitment, RT-PCR was performed. *RUNX2*, *ALP*, *PPARγ* and *FAPB4* markers were expressed in hPDLSCs maintained under differentiation (Figure 1C,D). Both mesengenic differentiations showed statistically significant differences between undifferentiated and differentiated cells.

### 3.4. Scanning Electron Microscopy Analysis and Immunofluorescence

**Figure 2:**
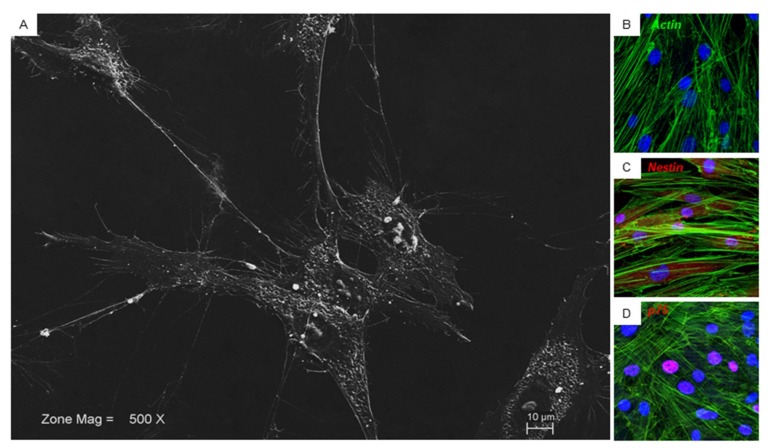
hPDLSCs morphological features. (**A**) Glass adherent hPDLSCs observed at SEM. (**B**) hPDLSCs observed at CLSM, green fluorescent stained the cytoskeleton actin. (**C**) hPDLSCs showed a positivity for nestin (red fluorescent). (**D**) hPDLSCs showed a positivity for p75 (red fluorescent). Nuclei were stained with TOPRO (blue fluorescent). Bar: 10 µm.

Glass adherent cells showed at SEM analysis a fibroblast like morphological shape with ovoidal nuclei and two or more nucleoli are visible. Long cytoplasmic processes make contact with neighboring cells (Figure 2A). Confocal microscopy observation showed the same morphological aspects (Figure 2B). Immunofluorescence of the hPDLSCs was performed in order to observe the expression of specific neurogenic markers. It showed a positivity for nestin at cytoplasmic level and for p75 localized at the nuclear level (Figure 2C,D).

### 3.5. Reactome and Pathway Analysis

**Figure 3:**
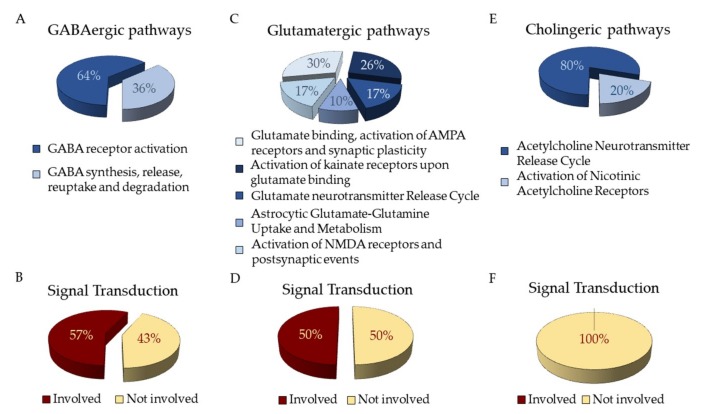
Distribution of genes express in hPDLSCs as identified in Reactome Database. (**A**) Distribution of genes related to GABAergic pathways (**B**) and their involvement in “Signal Transduction” pathway. (**C**) Distribution of genes related to Glutamatergic pathways (**D**) and their involvement in “Signal Transduction” pathway. (**E**) Distribution of genes related to Cholinergic pathways (**F**) and their involvement in “Signal Transduction” pathway.

The NGS analysis returns files in Fastq format containing the sequenced reads of our cDNA pool. The genes obtained after the NGS analysis were studied by the “Analysis” tool in the Reactome database that recognizes 2905 genes. Sixty-four of these genes are part of “Neuronal System” pathway. The analysis of hPDLSCs transcriptomic profile reveals genes related to ion channel receptors for GABA_B_ (*GABBR1* and *GABBR2*), acetylcholine (*CHRNA1*) and glutamate (GRINA) (Table 1). Genes are distributed as shown in Fig 3. The pathways related to GABA neurotransmitters are “GABA receptor activation” and “GABA synthesis, release, reuptake and degradation” (Figure 3A). Among those, 3 genes encoded for Adenylate Cyclase proteins act as Signal Transductors in the “Inhibition of adenylate cyclase pathways” that is directly linked to “Neuronal system”. (Figure 3B). The pathways related to glutamate are collected in “Glutamate binding, activation of AMPA receptors and synaptic plasticity”, “Activation of kainate receptors upon glutamate binding”, “Glutamate Neurotransmitter Release Cycle”, “Astrocytic Glutamate-Glutamine Uptake and Metabolism” and “Activation of NMDA receptors and postsynpatic events” (Figure 3C). Among those genes, 17 are involved in “Signal Transduction” (Figure 3D). The pathways related to acethylcoline are inside “Acetylcholine Neurotransmitter Release Cycle” and “Activation of Nicotinic Acetylcholine Receptors” (Figure 3E). None of the genes in these pathways is involved also in “Signal Transduction” (Figure 3F).

### 3.6. Western Blot Results

**Figure 4:**
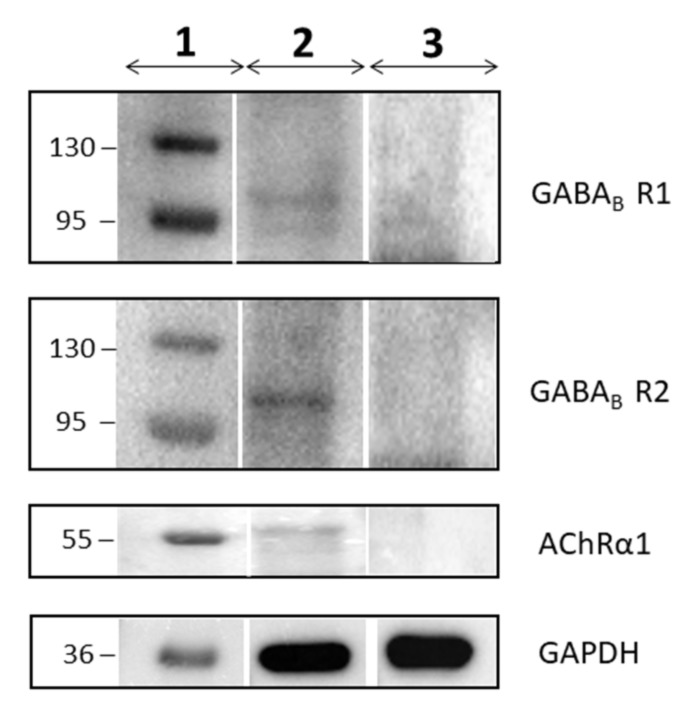
The image shows the Western Blot analysis for GABABR1, GABABR2, AChRα1 and GAPDH proteins about the hPDLSCs and the DPK-SKDF-H lineage. The column 1 represents the protein molecular weight markers, the column 2 the hPDLSCs and the column 3 the DPK-SKDF-H. The analysis shows the expression of the protein for each of them in hPDLSCs, but there is no expression in DPK-SKDF-H.

Western Blot analysis was used to confirm the expression of the GABA_B_ and Acetylcholine receptors. As shown in Figure 4, the proteic extract of hPDLSCs expresses GABA_B_R1, GABA_B_R2 and AChRα1. In contrast, the proteic extract of DPK-SKDF-H does not express these receptors. 

## 4. Discussion

**Figure 5:**
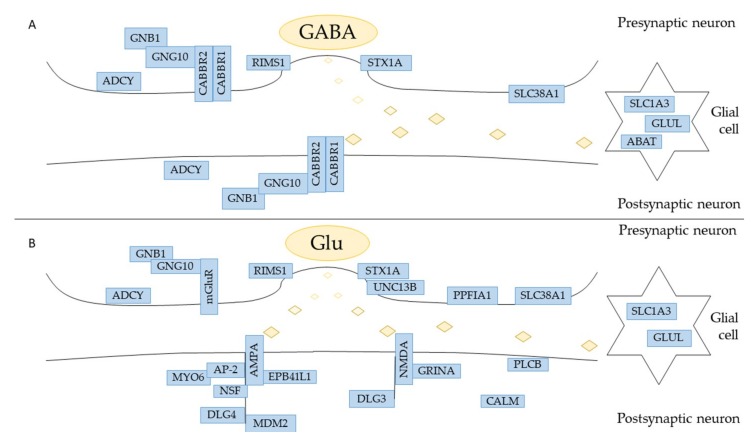
Representation of the synapsis in which are involved GABA (**A**) and glutamate (**B**) neurotransmitter. In the figure, all the genes that were found in hPDLSCs are highlighted. For a better reading, the genes *ADCY3*, *ADCY4* and *ADCY9* are clustered in “*ADCY*”. The genes *PLCB1* and *PLCB3* are grouped in “*PLCB*”. The genes *CALM1* and *CALM2* are bundled in “*CALM*”.

The etiopathogenesis of the neurodegenerative diseases is linked to the death of cells in nervous tissue. Their incidence is growing and stem cells are recently receiving attention from researchers because they can potentially replace damaged nervous cells. In particular, the hPDLSCs are becoming important because there is no ethical concern associated with their usage, they are easy to retrieve, derive from neural crest origin, and they can differentiate into mesengenic and neurogenic lineages. [28,29,30]. Indeed, the hPDLSCs cultured in basal medium spontaneously express neural markers as Nestin and GAP-43. Nestin is a protein belonging to class VI of intermediate filaments, expressed in stem/progenitor cells in the mammalian CNS during development [31,32]. In order to better understand the multipotent state of hPDLSCs, their physiological expression of ion channels receptors was investigated. The transcriptomic profile of the hPDLSCs was retrieved by three control individuals taking advantage of NGS technology. The RNA-seq analysis reveals 4378 genes express. In order to investigate all the genes involved in the nervous system, a database containing biological pathways (Reactome) was used. The “Neuronal System” pathway counts 64 genes. The hPDLSCs physiologically express the genes *GABBR1* and *GABBR2*, *GRINA*, *CHRNA1* related respectively to GABA_B_, NMDA and nACh receptors (Figure 5). For this reason, we studied all of the subpathways in which they are involved. A Western Blot analysis of hPDLSCs confirmed the expression of the GABA_B_R1, GABA_B_R2 and AChRα1 proteins. In order to verify whether the expression of *GABBR1* and *GABBR2* is specific to the hPDLSCs, the lineage of fibroblasts DPK-SKDF-H was used as control. Our results showed that DPK-SKDF-H does not express proteins for these receptors (Figure 4). *GABBR1* and *GABBR2* are indirectly involved with ion channel membrane proteins through the guanine nucleotide-binding proteins (G proteins). In hPDLSCs, *GNG10* and *GNB1* genes are expressed, that encode for two subunits of G proteins. In particular, *GNG10* encodes for a protein that directly interacts with *GABBR2* subunit [33]. The G proteins complex promotes the flow of potassium and calcium ions and acts as an inhibitor for Adenylate Cyclase proteins. The hPDLSCs express the *ADCY3*, *ADCY4* and *ADCY9* genes, that encode respectively for Adenylate Cyclase type 3, type 4 and type 9 that catalyze ATP in cyclic adenosine monophospate (cAMP) [34]. Moreover, the hPDLSCs express also *ABAT* and *GLUL* genes that encode for proteins involved in the catabolism of *GABA*. *ABAT* encodes for the *GABA*-transaminase enzyme that converts *GABA* into succinic semialdehyde. The *GLUL* gene encodes for the glutamine synthetase enzyme that synthesizes L-glutamine in L-glutamate. *SLC1A3* and *SLC38A1* encode for carrier proteins. Specifically, the *SLC1A3* gene encodes for amino acid transporter 1 protein, that is involved in the transport of the glutamate [35], whereas *SLC38A1* encodes for the sodium-coupled neutral amino acid transporter 1, that has high affinity for glutamine [36]. In hPDLSCs, we also find RIMS1 and STX1A that are involved in the exocytosis of *GABA*. *RIMS1* gene, that belongs to RAS superfamily, is involved in calcium channel modulation preparing the active zone of the synapsis for exocytosis [37]. The syntaxin-1A, protein encoded by STX1A, is characterized by a SNARE (SNAp REceptor) domain that allows the fusion to take place of the vesicles with the presynaptic membrane [38]. The hPDLSCs express also genes related to glutamate. Glutamate is a neurotransmitter that can be intercepted by metabotropic or ionotropic receptors.

Our results show that hPDLSCs only express the *GRINA* gene, a transmembrane NMDA subunit associated protein that binds the glutamate. Further, *PLCB1* and *PLCB3* are two genes that encode for Phospholipase C Beta proteins that allow to release the diacylglycerol (DAG), another secondary messenger [39]. The hPDLSCs express also several genes which encoded proteins are mainly located in the postsynaptic neurons. These proteins are involved in the maturation of the synapses and in the trafficking of the neurotransmitter. In hPDLSCs they are encoded by: AP-2 complex (*AP2A1*, *AP2A2*, *AP2M1*, *AP2S1*), *MYO6*, *NSF*, *EPB41L1*, *CALM1*, *CALM2*, *DLG3*, *DLG4*, *MDMD2*. The AP-2 complex, supported by the unconventional myosin VI (encoded by *MYO6*), reduces the excitatory stimuli by endocytosis of AMPA [40]. The NSF gene encodes for the Vesicle-Fusing. ATPase binds the subunit GluA2 of the AMPA receptor in order to facilitate the fusion of the membrane and the vesicle by the SNARE domain [41]. The trafficking of the receptor AMPA is also regulated by Band 4.1-like protein 1, encoded by *EPB41L1* gene, that handles the long-term potential in the synapses mainly by palmitoylation and phosphorylation on GluR1 subunit [42]. Calmodulins are calcium-modulated proteins encoded by genes like *CALM1* and *CALM2*. They are involved in the calcium homeostasis carrying it towards the endoplasmic reticulum or out of the cell [43]. *DLG3* encodes for the protein Disks Large homolog 3 that stabilizes *NMDA* receptor [44] while *DLG4* encodes for the protein Disks Large homolog 4 that interacts also with *AMPA* receptor [45]. The hPDLSCs express also *MDM2*, encoding for an E3 ubiquitin ligase, that indirectly causes the *AMPA* receptor endocytosis [46]. The transcriptomic profile of hPDLSCs also reveals a gene encoded for proteins located on presynaptic neurons like PPFIA1 and UNC13B. The *PPFIA1* gene, encoded for Liprin-Alpha-1 protein, belongs to the protein family of liprins that is localized closed to cell focal adhesions. Liprins are involved in functional plasticity, the formation of the synapses and in axon guidance [47]. UNC13B is characterized by a SNARE domain that allows the docking to take place among the vesicle and the membrane aimed at the release of glutamate [48]. Only *CHRNA1* gene, encoding for one of the five subunit of nAChr, is expressed in cholinergic synapses [49].

## 5. Conclusions

The RNA-seq analysis performed in this study reveals the expression of genes that encode for ionotropic and metabotropic receptors, along with proteins located in presynaptic and postsynaptic neurons. The hPDLSCs are known to differentiate in different neuronal cells. In our results, the expression of GABA_B_R1, GABA_B_R2 and AChRα1 suggests that a subpopulation of hPDLCSs differentiate in neurons.

## Figures and Tables

**Table 1:** Genes of our dataset included in Cholinergic, GABAergic, Glutamatergic REACTOME pathways.

Genes	Protein Name (Uniprot)	Pathways	Signal Transduction
*ABAT*	4-Aminobutyrate Aminotransferase	GABA	No
*ADCY3*	Adenylate Cyclase type 3	GABA, GL	Yes
*ADCY4*	Adenylate Cyclase type 4	GABA, GL	Yes
*ADCY9*	Adenylate Cyclase type 9	GABA, GL	Yes
*AP2A1*	AP-2 complex subunit Alpha 1	GL	Yes
*AP2A2*	AP-2 complex subunit Alpha 2	GL	Yes
*AP2M1*	AP-2 complex subunit Mu 1	GL	Yes
*AP2S1*	AP-2 complex subunit Sigma 1	GL	Yes
*CALM1*	Calmodulin-1	GL	Yes
*CALM2*	Calmodulin-2	GL	Yes
*CHRNA1*	Acetylcholine Receptor subunit Alpha	CH	No
*DLG3*	Disks Large homolog 3	GL	Yes
*DLG4*	Disks Large homolog 4	GL	Yes
*EPB41L1*	Band 4.1 Like protein 1	GL	No
*GABBR1*	Gamma-Aminobutyric Acid Type B Receptor Subunit 1	GABA	Yes
*GABBR2*	Gamma-Aminobutyric Acid Type B Receptor Subunit 2	GABA	Yes
*GLUL*	Glutamine synthetase	GABA, GL	No
*GNB1*	Guanine Nucleotide-binding protein G(I)/G(S)/G(T) subunit beta-1	GABA, GL	Yes
*GNG10*	Guanine Nucleotide-binding protein G(I)/G(S)/G(O) subunit gamma-10	GABA, GL	Yes
*MDM2*	E3 ubiquitin-protein ligase Mdm2	GL	Yes
*MYO6*	Unconventional myosin-VI	GL	No
*NSF*	Vesicle-fusing ATPase	GL	No
*PLCB1*	1-phosphatidylinositol 4,5-bisphosphate phosphodiesterase beta-1	GL	Yes
*PLCB3*	1-phosphatidylinositol 4,5-bisphosphate phosphodiesterase beta-3	GL	Yes
*PPFIA1*	Liprin-Alpha-1	CH, GL	No
*RIMS1*	Regulating Synaptic Membrane Exocytosis 1	CH, GABA, GL	No
*SLC1A3*	Excitatory amino acid transporter 1	GABA, GL	No
*SLC38A1*	Sodium-coupled neutral amino acid transporter 1	GABA, GL	No
*STX1A*	Syntaxin-1A	CH, GABA, GL	No
*UNC13B*	Protein Unc-13 Homolog B	GL, CH	No

The acronym CH, GABA and GL stand respectively for Cholinergic, GABAergic and Glutamatergic pathways.
